# Sequence Determination of *Burkholderia pseudomallei* Strain NCTC 13392 Colony Morphology Variants

**DOI:** 10.1128/genomeA.00925-13

**Published:** 2013-11-07

**Authors:** Julia Vipond, Jennifer Kane, Graham Hatch, Jamison McCorrison, William C. Nierman, Liliana Losada

**Affiliations:** Public Health England, Salisbury, United Kingdoma; The J. Craig Venter Institute, Rockville, Maryland, USAb; The George Washington University, Washington, DC, USAc

## Abstract

*Burkholderia pseudomallei* is a biothreat and the causative agent of melioidosis. There are at least seven known colony morphotypes of *B. pseudomallei* that appear to have different virulence properties in animal models. We report the genome sequence of *B. pseudomallei* strain NCTC 13392 and the genomic variations of its eight morphotype derivatives.

## GENOME ANNOUNCEMENT

*Burkholderia pseudomallei* bacteria can be found in a wide variety of habitats and are capable of infecting a large range of hosts. Recent reports have documented numerous colony morphology types, even within a clonal population, and have indicated that differences exist in the proteomes and the virulence properties of these variants (1, 2). To understand the genetic underpinnings of these differences, we sequenced and assembled the genome of the wild-type morphology variant of *B. pseudomallei* strain NCTC 13392. In addition, we sequenced eight other morphotypes derived from the same strain after passage in a small animal model system.

All animal work in the United Kingdom is conducted in compliance with the UK Home Office Animals (Scientific Procedures) Act 1986. In addition, all procedures were conducted under a project license approved by the ethical review process of Public Health England, Salisbury, United Kingdom, prior to Home Office approval.

BALB/c mice were infected with a presented dose of approximately 3,000 CFU via aerosol challenge using the AeroMP-Henderson apparatus. After 1 to 4 days, depending on the health of the animals, solid organs were homogenized in Luria-Bertani broth (LB) and bacteria were plated on selective Ashdown’s medium and incubated for 7 days at 37°C (3). Colonies displaying variant morphotypes were picked and genomic DNA was prepared with the DNeasy blood and tissue kit (Qiagen) according to the manufacturer’s instructions. Paired-end 100-bp Illumina fragment reads (~60× coverage) were used for genome sequence determination. All reads were used to generate assemblies with Celera Assembler 6.1 (4). The resulting assemblies ranged from 61 to 80 scaffolds containing 143 to 263 contigs, with N_50_s ranging from 52 kb to 91 kb. As seen previously with other *B. pseudomallei* strains, all genomes had 2 chromosomes (5, 6) with sizes similar to those of the reference strain *B. pseudomallei* K96243 (roughly 4.1 Mb and 3.17 Mb for chromosomes 1 and 2, respectively). The genome sequence of the isolate with wild-type morphology was annotated using the J. Craig Venter Institute (JCVI) annotation pipeline (http://www.jcvi.org) and submitted to GenBank.

As expected, the genomes were >99% identical between all the isolates. The sequence reads from eight other morphotypes were mapped to the wild-type morphology reference genome. In each case, nearly 100% of the bases were covered by sequencing. Single nucleotide polymorphisms (SNP), insertions/deletions (indels), and structural variations were identified using the CLC Genomics Workbench. After filtering, no single-nucleotide polymorphisms (SNPs) were detected in any of the morphotypes. Since other organisms may regulate morphology by phase variation via strand slippage at repetitive sequences (7), we performed a comparative analysis of short tandem repeats in the morphotypes. Though we detected differences in structural variants and/or variations in numbers of tandem repeats in the morphotypes, none of the differences were conserved across morphotypes and thus might be a result of differences in sequencing and assemblies rather than of biological differences. The results presented here suggest that the differences in morphology are more likely due to epigenetic factors, gene expression programs, and/or proteome variations rather than a result of genomic sequence differences.

### Nucleotide sequence accession numbers.

The *B. pseudomallei* whole-genome shotgun project has been deposited at GenBank under the accession no. AUVI00000000, AWEO00000000, AUVJ00000000, AUVK00000000, AUVL00000000, AUVM00000000, AUVN00000000, AUVO00000000, and AUVP00000000.
